# Coronavirus disease 2019 and sudden sensorineural hearing loss

**DOI:** 10.1017/S0022215120002145

**Published:** 2020-10-01

**Authors:** B Lang, J Hintze, B Conlon

**Affiliations:** Department of Otorhinolaryngology and Head and Neck Surgery, St James University Hospital, Dublin, Ireland

**Keywords:** Severe Acute Respiratory Syndrome, Covid-19, Coronavirus, Sensorineural Hearing Loss, Steroids

## Abstract

**Background:**

Severe acute respiratory syndrome coronavirus 2 emerged in December 2019 in Wuhan, China, and was declared a global health emergency of international concern by the World Health Organization on 30th January 2020. It has resulted in almost 600 000 deaths to date worldwide. Sudden sensorineural hearing loss is a known complication of a number of viral infections, but there is little in the literature to date on its association with coronavirus disease 2019.

**Case report:**

This paper presents the case of a 30-year-old female staff nurse who contracted coronavirus disease 2019 and presented to our department with a significant unilateral sensorineural hearing loss confirmed on audiogram. She was treated with a course of oral steroids, but unfortunately there was no improvement in her hearing.

**Conclusion:**

This case report is important as it highlights the importance of having a low index of suspicion when patients present with a variety of symptoms not previously associated with coronavirus disease 2019. The paper also discusses the controversy surrounding the use of steroids in the management of this disease.

## Introduction

The first cases of coronavirus disease 2019 (Covid-19) likely occurred from a zoonotic transmission in China in December 2019, linked to a large seafood market that also traded in live wild animals.^1^ The causative virus, severe acute respiratory syndrome coronavirus 2 (SARS-CoV-2), is capable of human-to-human transmission via droplet and direct contact. The virus spread rapidly to other parts of China and then to other locations.

A wide range of clinical features may appear 2–14 days after exposure to the virus. The most common symptoms include fever, cough, shortness of breath, sore throat, headache, muscle pain, and taste and smell disturbance. Elderly and immunocompromised individuals appear to be more susceptible to severe infection, which can lead to acute respiratory distress syndrome, multiorgan failure and death.^2,3^

Sudden sensorineural hearing loss (SNHL) is a known complication of a number of viral infections, but there is little in the literature to date on its association with Covid-19.

## Case report

We present the case of a 30-year-old female staff nurse who was working in a university tertiary hospital earlier during the Covid-19 pandemic. Written informed consent was obtained from the patient.

She initially developed symptoms on 19th April 2020 in the form of fever, cough, headache and myalgia. Severe acute respiratory syndrome coronavirus 2 was detected in a nasopharyngeal swab on 20th April 2020. She self-isolated at home and did not require admission to hospital. By 17th May, all her symptoms had resolved, but she then developed sudden right-sided hearing loss and tinnitus. She had no otalgia, otorrhoea or vertigo, and had no history of head trauma. She was otherwise fit and healthy, and was not taking any regular medications.

**Fig. 1.**
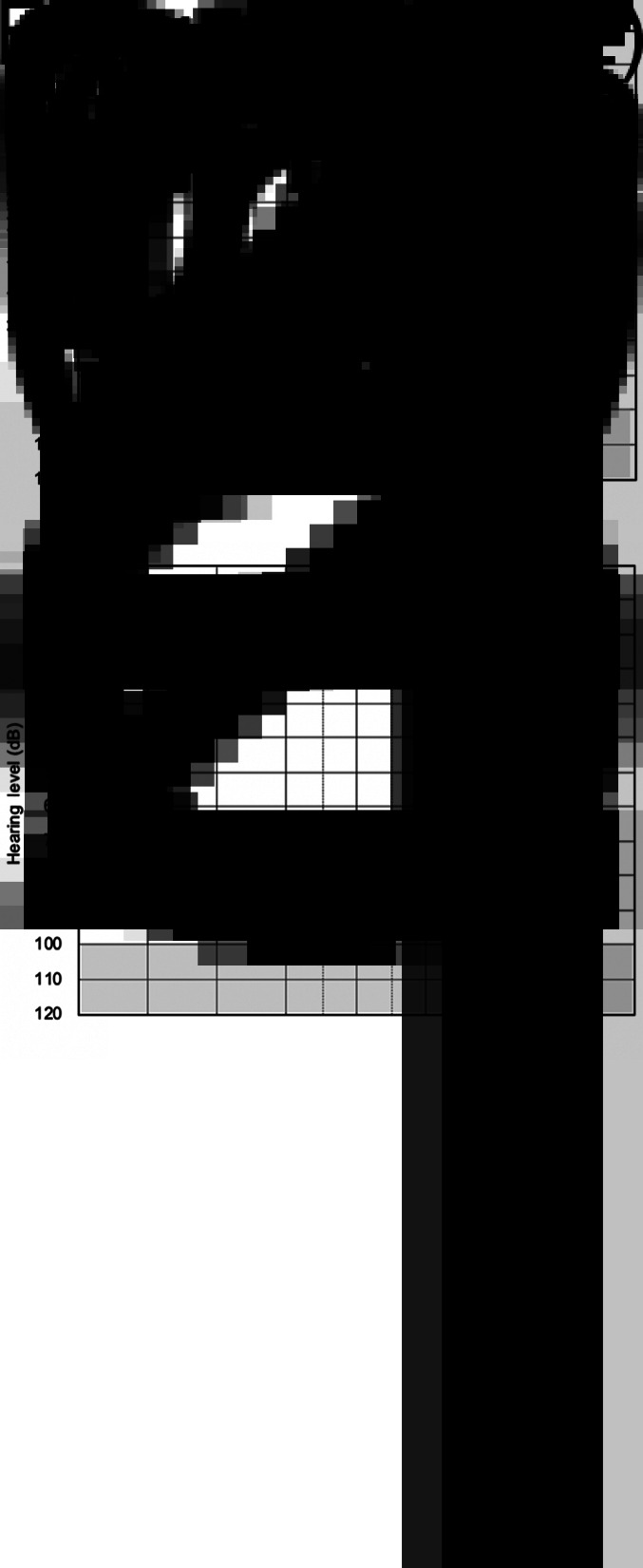
Initial audiograms for (a) right ear and (b) left ear.

Her right-sided hearing loss and tinnitus persisted. She was seen by the occupational health department and subsequently referred to the ENT out-patients department. She was seen in the ENT department on the 9th June; otoscopic examination findings were normal. Audiology testing confirmed a profound high-frequency SNHL (Figure 1).

**Fig. 2.**
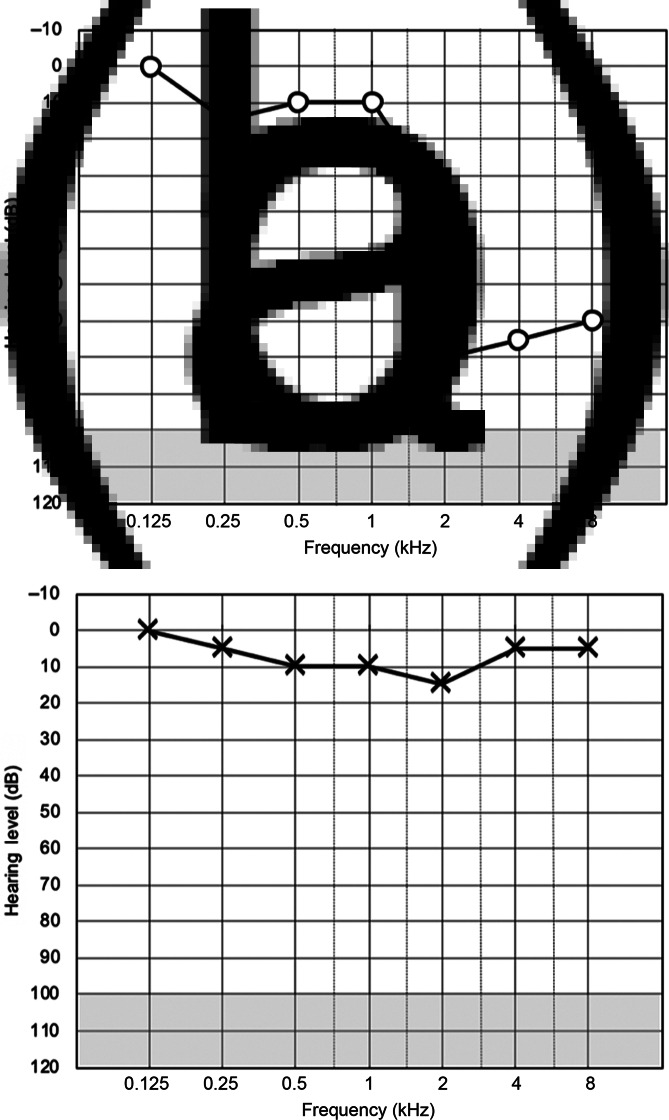
Follow-up audiograms for (a) right ear and (b) left ear.

Although it had been seven weeks since the detection of SARS-CoV-2 and three weeks following the hearing loss onset, the decision was made to begin a trial of oral steroids. She was commenced on a tapering dose of oral prednisolone. Audiological assessment was repeated one week later and unfortunately there was no improvement (Figure 2).

She underwent magnetic resonance imaging of the brain, the findings of which were normal, with no abnormalities at the internal auditory meatus.

## Discussion

Severe acute respiratory syndrome coronavirus 2 emerged in December 2019 in Wuhan, China, and was declared a global health emergency of international concern by the World Health Organization on 30th January 2020. It has resulted in almost 600 000 deaths to date worldwide.

Sudden SNHL is defined as SNHL of 30 dB or more in at least three consecutive frequencies over a 3-day period. A number of mechanisms have been implicated: neuritis caused by viral involvement of the cochlear nerves, cochleitis due to viral involvement of the cochlea and peri-lymphatic tissues, and the stress response resulting in the cross-reaction of the inner-ear antigens to viral infections.^4^

There are only three other case reports to our knowledge that have reported sudden SNHL in Covid-19 positive patients.^3–5^ Immune-mediated inflammation likely plays a significant role given that severe cases of Covid-19 have been associated with dysregulation of the immune system. In these severe cases, an increased neutrophil-to-lymphocyte ratio and elevated inflammatory cytokines such as interleukin 6 have been observed.^5^ In addition, Covid-19 has been shown to have deleterious effects on cochlear hair cells, with reduced amplitude of transient-evoked otoacoustic emissions in a group of 20 coronavirus positive asymptomatic patients.^6^

Corticosteroids are widely used in the treatment of sudden SNHL; however, steroids are controversial in the setting of Covid-19. Initially, there were reports that steroids may delay viral clearance and increase infection severity.^4,7,8^ Kilic *et al*. reported on five cases of sudden SNHL during the coronavirus pandemic.^4^ The one patient who was Covid-19 positive was treated with oral hydroxychloroquine, while the remaining four coronavirus negative patients received standard care with corticosteroids. The Covid-19 positive patient had complete recovery of hearing at one month. Two of the remaining Covid-19 negative patients had complete or partial recovery.

- Severe acute respiratory syndrome coronavirus 2 emerged in December 2019 and was declared a global health emergency in January 2020
- Common symptoms include fever, cough, shortness of breath, sore throat, headache, muscle pain, and taste and smell disturbance
- Sudden sensorineural hearing loss (SNHL) is a complication of some viral infections, but its relationship with coronavirus disease 2019 (Covid-19) is unknown
- This paper reports on a 30-year-old staff nurse who contracted Covid-19 and presented with unilateral SNHL
- Although controversial in Covid-19, the patient was treated with oral steroids, but there was no improvement in hearing
- A low index of suspicion is important when patients present with symptoms like sudden SNHL not previously widely associated with Covid-19

In addition, two commentaries published in *Lancet* journals in February and March 2020, respectively, reported that corticosteroids should not be used for the treatment of Covid-19 as they have no mortality benefit, they delay clearance of viral RNA and they have serious side effects.^8,9^ However, this assumption was based on the findings of previous studies indicating that steroids increase mortality in patients with influenza and other viruses.

In March 2020, the Randomised Evaluation of Covid-19 Therapy (‘RECOVERY’) trial, led by scientists from the University of Oxford, was established to test a range of potential treatments for Covid-19. Over 11 000 hospitalised patients with Covid-19 were enrolled. On 16th June 2020, it was reported at a press release that dexamethasone reduced deaths by one-third in ventilated patients and by one-fifth in patients receiving oxygen only. Steroids had no effect on patients with mild Covid-19 who were not receiving oxygen.^10^

Given the controversy surrounding steroid use in Covid-19, one could consider intratympanic steroids. In the setting of sudden SNHL, intratympanic steroids have been used as a primary monotherapy, combined with oral steroids or used as a salvage treatment if initial treatment has failed. Studies have shown minimal systemic absorption of intratympanic steroids, with reduced risk of systemic side effects, in addition to much higher concentrations of steroid in the cochlear perilymph. This would make it a good alternative treatment for Covid-19 related sudden SNHL until we have more literature on the use of steroids in patients with SARS-CoV-2.^11^

## Conclusion

It is important to have a low index of suspicion for patients presenting with a wide variety of symptoms during the Covid-19 pandemic, in order to break the cycle of transmission and prevent the spread of infection. Steroids in this setting are controversial and there have been mixed reports in the literature. They appear to reduce deaths in those patients with severe Covid-19 with acute respiratory distress syndrome, but their use in patients with mild disease or sudden SNHL remains in doubt.
